# An app-, web- and social support-based weight loss intervention for adults with obesity: the ‘HelpMeDoIt!’ feasibility randomised controlled trial

**DOI:** 10.1186/s40814-020-00656-4

**Published:** 2020-09-19

**Authors:** Sharon Anne Simpson, Lynsay Matthews, Juliana Pugmire, Alex McConnachie, Emma McIntosh, Elinor Coulman, Kathryn Hughes, Mark Kelson, Sarah Morgan-Trimmer, Simon Murphy, Olga Utkina-Macaskill, Laurence Anthony Russell Moore

**Affiliations:** 1 MRC/CSO Social and Public Health Sciences Unit, Institute of Health and Wellbeing, University of Glasgow, Berkeley Square, 99 Berkeley Street, Glasgow, G3 7HR UK; 2 Robertson Centre for Biostatistics, Institute of Health and Wellbeing, University of Glasgow, Robertson Centre, Boyd Orr Building, Glasgow, G12 8QQ UK; 3 Health Economics and Health Technology Assessment Unit (HEHTA), Institute of Health and Wellbeing, University of Glasgow, 1 Lilybank Gardens, Glasgow, G12 8RZ UK; 4 Centre for Trials Research, Cardiff University, Neuadd Meirionnydd, Heath Park Way, Cardiff, CF14 4YS UK; 5 Division of Population Medicine, School of Medicine, Cardiff University, Neuadd Meirionnydd, Heath Park Way, Cardiff, CF14 4YS UK; 6 College of Engineering, Mathematics and Physical Sciences, School of Mathematics/The Alan Turing Institute, University of Exeter, Harrison Building, Streatham Campus, North Park Road, Exeter, EX4 4QF UK; 7 Institute of Health Research, College of Medicine and Health, University of Exeter, College House, St Luke’s Campus, Heavitree Road, Exeter, EX1 2LU UK; 8 Centre for the Development and Evaluation of Complex Interventions for Public Health Improvement (DECIPHer), Cardiff School of Social Sciences, Cardiff University, Cardiff, CF10 3AT UK

**Keywords:** Digital health, Obesity, Weight loss, Social support, Social network, Goal setting, Self-monitoring, Physical activity, Diet

## Abstract

**Background:**

Social support has an important role in successful weight loss. The aim of this study was to assess the feasibility and acceptability of an app-, web- and social support-based intervention in supporting adults with obesity to achieve weight loss.

**Methods:**

The intervention and evaluation methods were tested in a feasibility randomised controlled trial. Adults in the Greater Glasgow and Clyde Health Board area of Scotland with a body mass index ≥ 30 kg/m^2^ were recruited and randomised 2:1 (intervention to control). The feasibility and acceptability of the intervention and trial methods were assessed against pre-specified progression criteria, via process, economic and outcome evaluation. Three primary outcomes were explored: BMI, diet and physical activity, as well as a number of secondary outcomes. The intervention group had access to the HelpMeDoIt! intervention for 12 months. This encouraged them to (i) set goals, (ii) monitor progress and (iii) harness social support by inviting ‘helpers’ from their existing social network. The control group received a healthy lifestyle leaflet.

**Results:**

One hundred and nine participants were recruited, with 84 participants (77%) followed-up at 12 months. The intervention and trial methods were feasible and acceptable. Participants and helpers were generally positive. Of the 54 (74%) participants who downloaded the app, 48 (89%) used it. Interview data indicated that HelpMeDoIt! promoted social support from existing social networks to support weight loss. This support was often given outside of the app.

Outcomes were compared using linear regression models, with randomised group, the baseline measurement of the outcome, age and gender as predictor variables. These analyses were exploratory and underpowered to detect effects. However, all pre-specified primary outcome effects (BMI, diet and physical activity) had wide confidence intervals and were therefore consistent with clinically relevant benefits. Objective physical activity measures perhaps showed most potential (daily step count (*p* = 0.098; 1187 steps [− 180, 2555])) and sedentary time (*p* = 0.022; − 60.8 min [− 110.5, − 11.0]). However, these outcomes were poorly completed.

**Conclusions:**

The study demonstrated that a novel social support intervention involving support from participants’ close social networks, delivered via app and website, has potential to promote weight loss and is feasible and acceptable.

**Trial registration:**

ISRCTN, ISRCTN85615983. Registered 25 September 2014

## Key messages regarding feasibility

1. What uncertainties existed regarding the feasibility?
  Was our web- and app-based healthy lifestyle intervention, that harnessed social support from friends and family, feasible to deliver and acceptable to participants?
  Would the intervention allow participants to engage social support and would this help them with weight-related lifestyle changes?
  Would our evaluation methods be feasible in a larger trial?
2. What are the key feasibility findings?
  The intervention was feasible to deliver and acceptable to participants.
  The social support aspect of the intervention was well-received and considered beneficial.
  Our evaluation methods would be feasible in a larger trial
3. What are the implications of the feasibility findings for the design of the main study?
  That this novel intervention involving social support from participants’ close social networks has potential to promote weight loss and is feasible and acceptable.

## Background

Poor diet, physical inactivity and high body mass index (BMI) have been highlighted in the top 10 risk factors for global burden of disease [1]. Preventive interventions which are engaging and can reach large numbers of people, including the underserved, are needed. Digital technologies have significant potential for engaging people with health behaviour change. Recent reports show that most adults in the UK, including those in socially disadvantaged groups, own a smartphone [2, 3].

The role of social support from family and friends is known to be important for successful weight loss and maintenance [4, 5]. Family and friends are significant social influences on health behaviours due to factors such as intimacy, influence and proximity to day-to-day health behaviours. They are likely to be especially important at times of potential relapse. Many apps and websites have chat forums or communities of support from fellow users. There are also apps that can help connect a user with family members or friends to share stats on step count, exercise sessions, or other competitive endeavours. However, none of these apps are specifically designed to get users to engage with those closest to them to help support them in losing weight by checking on their progress and providing encouragement. This is despite existing evidence indicating the positive role of family and friends in promoting effective behaviour change rather than anonymous online contacts [6, 7]. As far as we were aware none of these sites or apps offered the combination of elements that we used in HelpMeDoIt!, most importantly user-nominated social support from key individuals within that person’s social network, i.e. existing friends and family who will in many cases be the people that participants eat and exercise with.

There is also strong evidence for goal setting and self-monitoring as successful behaviour change techniques [8–10]. These techniques derive from social cognitive theory [11] and control theory [12], the key psychological theories which inform the HelpMeDoIt! intervention, alongside social support theories. Combining social support with these techniques, using accessible, engaging technology, has the potential to impact behaviour change at a population level for low cost [13, 14]. If brief engagement with an app could mobilise existing social connections to support longer term change, then this could offer a sustainable approach to weight loss and maintenance. The main aim of the study is to explore the feasibility, acceptability and impact of a weight loss intervention, delivered via smartphone app and website, in supporting adults with obesity to achieve weight loss goals, and further to identify the value and optimal design of a future effectiveness trial.

## Objectives

1. To explore the feasibility and acceptability of the intervention and its potential to reach traditionally underserved groups (e.g. lower socioeconomic groups)
2. To explore the barriers and facilitators to implementing the intervention
3. To investigate how participants and helpers engage with goal setting, monitoring and social support using new technologies and how these elements interact within a behaviour change intervention
4. To test the logic model and theoretical basis of the intervention
5. To investigate recruitment and retention and assess the feasibility and acceptability of outcome measures for diet and physical activity in this population
6. To use outcome data (diet, physical activity, BMI) to help decide on a primary outcome and to inform the calculation of an appropriate sample size for a full trial
7. To assess data collection tools and obtain estimates of key cost drivers to inform the design of a future cost-effectiveness analysis
8. To assess whether an effectiveness trial is warranted

## Methods

### Design

The study was completed in two stages: intervention development and early testing (stage 1) and a feasibility randomised controlled trial (stage 2).

#### Stage 1

**Fig. 1:**
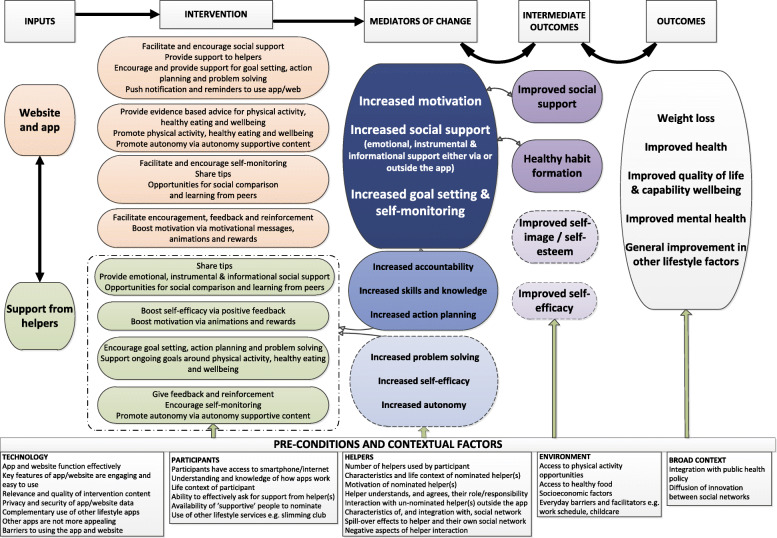
The logic model

Over a 12-month period, we developed and piloted the intervention. The intervention was developed iteratively with involvement of a panel of user representatives (*n* = 10) and a user testing group (*n* = 28), who were recruited via advertising in local organisations, Gumtree, Twitter and Facebook. We purposively recruited a varied group in terms of age, gender, socioeconomic status and experience of using apps. All participants wanted to lose weight. Working closely with these groups, we considered how to promote engagement as well as increase the acceptability and functionality of the app and website. This early intervention development work is detailed in our full monograph [15]. We followed the UK Medical Research Council (MRC) guidance [16] and the 6SQUID [17] steps and adopted a person-centred approach [18]. At the end of the development process, we produced an updated programme theory and logic model [15] (Fig. 1 shows the final logic model from the end of the stage 2). Logic model components were directly linked to the app and website components [19], e.g. to boost *motivation* participants could see progress graphs, they could receive in-app medals and trophies for progress and most importantly encouragement from their helpers.

#### Stage 2

Stage two was a feasibility RCT, with a process evaluation and health economic component, which aimed to examine feasibility, acceptability and trial parameters for a future trial.

### Participants

Participants in the Greater Glasgow and Clyde Health Board area of Scotland were eligible if they were aged 18–70, had a body mass index (BMI) of ≥ 30 kg/m^2^, owned a smartphone and were interested in losing weight. The study had several exclusion criteria listed below:

- Terminal illness
- Previous bariatric surgery
- Dementia
- Pregnancy
- Low competence in English (inability to complete study materials)
- Contraindications to physical activity
- Participant in stage 1 intervention development
- Being a nominated helper in the trial

#### Sample size

This was a feasibility study and thus the main focus was to assess the acceptability of the intervention, the feasibility of the evaluation methods and to estimate parameters for a larger study. We intended to recruit 120 participants and estimated a dropout rate of 30%. The final sample size of 84 for analysis was not powered to detect differences between groups for the proposed effectiveness outcomes (BMI, physical activity and diet). However, it allowed estimation of any feasibility proportion (e.g. proportions retained/found the study acceptable/provided outcome data) across the whole sample with a 95% confidence interval of plus or minus 11 percentage points. This would also allow for the estimation of the mean of a continuous outcome (such as BMI) in the intervention arm with a 95% confidence interval of 0.262 of a standard deviation.

### Recruitment and randomisation

We recruited participants in the Greater Glasgow and Clyde Health Board area of Scotland between April and October of 2016 from three sources: primary care (searched primary care databases and sent letters to potentially eligible patients), online (e.g. Gumtree, Facebook and Twitter) and community settings (e.g. advertised via local press, slimming clubs, weight management clinics, study posters in community locations) [19].

Participants were screened for eligibility and a face-to-face appointment was arranged for consent and baseline data collection with a field worker. Since we were most interested in the acceptability of the intervention and to improve trial efficiency, we randomised participants using a 2:1 ratio planning for 80 participants in the intervention group and 40 in the control group. Participants were allocated using a mixed randomisation/minimisation algorithm to ensure balance with respect to gender and BMI (< 40, ≥ 40 kg/m^2^).

### Interventions

The intervention group were given access to the HelpMeDoIt! app and website for 12 months. The website provided evidence-based information on weight loss, setting and monitoring goals, as well as advice on harnessing social support from family and/or friends. The app allowed participants to (i) set goals for weight loss, (ii) monitor progress and (iii) invite one or more helpers from their existing social network. This is the key novelty of this intervention whereby the app aimed to mobilise social support from people in participants’ close social networks who are likely to have greater and sustained influence than, for example, those in online networks or weight loss groups. Helpers who agreed to provide support were also able to access the website and app and see participants’ goals and progress. They could provide support to the participant via the app and outside of the app (e.g. face-to-face, phone call). Further details of the intervention can be found in our published protocol paper [19]. The control group received a leaflet on healthy lifestyle and were offered access to the app and website after follow-up was complete. All participants were advised that they could continue to access other sources of lifestyle change/support such as attending weight loss groups and fitness classes.

### Outcomes

The key outcome of the study was whether pre-specified progression criteria were met in order to progress to a definitive trial. Feasibility and acceptability of the intervention and evaluation methods were assessed using the progression criteria outlined in Table 1 [15]. These criteria were approved by the Trial Steering Committee prior to Stage 2. Data were collected at baseline and 12 months and included (i) quantitative outcomes assessing three primary outcomes (BMI, physical activity and diet); (ii) secondary outcomes of weight, waist and hip circumference, social support, self-efficacy, motivation, mental health and health-related quality of life; (iii) qualitative interviews with participants and helpers at 6 and 12 months; (iv) health economic evaluation including measurement and valuation of NHS resource use, participant-borne costs, intervention costs and health-related quality of life and capability wellbeing. The feasibility measures and exploratory outcome measures used are detailed in Table 2. Three primary outcomes were assessed for use in a future effectiveness trial: body mass index (BMI), physical activity and diet. We explored which of these was most feasible by assessing acceptability and data completeness. Since measuring diet [34] and physical activity [35] in community-based trials is challenging, we assessed two ways of measuring these outcomes: the DINE questionnaire [21] and 24-h dietary recall [36] for measuring diet and accelerometer [22] and 7-day physical activity recall [37] for measuring physical activity (see published protocol for details) [19].

**Table 1:** Progression criteria from feasibility to full randomised controlled trial [15]

Progression *c*riterion	Method of assessment
1. *Are appropriate and effective routes of recruitment available to achieve a powered sample size in a full trial?*	− Coming close to the sample size, as judged by the Trial Steering Committee, with reasonable expectations of being able to address any recruitment issues
2. *Are participants willing to be randomised to the intervention?*	− Recruitment experiences of the study team and fieldworkers − Insight from qualitative interviews with participants
3. *Are appropriate retention rates achieved at 12-month follow-up?*	− Measured using the following scale in both the intervention and control group at 12-months: If ≥ 70% followed-up proceed; if 50–69% followed-up discuss with Trial Steering Committee; if ≤ 49% followed-up do not proceed
4. *Is the intervention feasible to deliver and acceptable to participants and their helpers?*	− USE questionnaire − Participant/helper interviews
5. *Do the majority (> 50%) of participants within the intervention group visit the app at least twice OR do 25% of participants randomised use it three or more times?*	− App usage statistics
6. *Are identified barriers and challenges to implementation of the intervention planned for and surmountable?*	− Process evaluation which will present a SWOT analysis and action plan
7. *Do the data collection procedures effectively collect the data required for a full trial? Successful completion of at least one data collection outcome measure (BMI, physical activity or healthy eating) at both baseline and at 12 months in those retained measured using the following scale:*	− If > 90% *of at least one* data collection measure completed proceed; − If 70–89% *of at least one* data collection measure completed discuss strategies for improvement in future trial with Trial Steering Committee; − If < 70% *of all three* data collection measures completed do not proceed without further modification and pilot
8. *Are the intervention costs of a full trial covered?*	− Identification of a source to pay access and treatment costs

**Table 2:** Feasibility measures and exploratory outcomes [15]

Measure	Method of measurement	Time-point
Demographics		
Case Report Form: gender, age, socioeconomic status, employment and education status, current weight loss status, current health status, current computer and phone use		Baseline and 12 months
Feasibility measures (reflecting progression criteria)		
Recruitment	Sample size and rate of recruitment, sources of recruitment	Post baseline
Randomisation	Interviews with participants and insight from study team	6 months
Retention	Retention rates for data collection at 12 months follow-up	12 months
Feasibility of app/website (intervention)	Interviews with participants, interviews with helpers, app and website usage statistics, USE [20] questionnaire	6 and 12 months, 12 months, 12 months and 12 months
Data collection	Rates of completion for different measures	Baseline and 12 months
Exploratory primary outcomes		
BMI (kg/m^2^)	Physical measurement of height (m) and weight (kg)	Baseline and 12 months
Diet	DINE questionnaire [21] (via telephone), 4 days of 24-h dietary recall	Baseline and 12 months
Physical activity	7 day accelerometry [22], 7 day Physical Activity Recall Questionnaire [23]	Baseline and 12 months
Secondary outcomes		
Anthropometric changes	Waist and hip circumference (cm)	Baseline and 12 months
Health-related quality of life	EQ5D 3 -L questionnaire [24], ICECAP A scale [25]	Baseline and 12 months
Mental health	General Health Questionnaire (GHQ12) [26]	Baseline and 12 months
NHS resource use and participant-borne costs	Specially designed resource use questionnaire	Baseline and 12 months
Social support	Exercise and Eating Habits Social Support Scales [27]	Baseline and 12 months
Self-efficacy	Weight [28] and Exercise Efficacy Lifestyle Scales [29, 30]	Baseline and 12 months
Motivation	Treatment Self-Regulation Questionnaire [31]	Baseline and 12 months
Smoking use	Heaviness of Smoking Index (HIS) [32]	12 months
Alcohol use	Alcohol Use Disorders Identification Test (AUDIT-C) [33]	12 months

### Assessment of harms

We developed a standardized operating procedure for dealing with any reported adverse events, advised participants to discuss any health concerns with their GP and encouraged participants and fieldworkers to report negative outcomes to the study team. In addition, as part of the qualitative interviews, we explored the issue of ‘harm’. The intervention was low risk to participants.

### Process evaluation

The process evaluation focussed on issues related to the intervention including; context, fidelity, exposure, reach and the programme theory and logic model. Both quantitative (app and web usage statistics) and qualitative data (interviews) informed the process evaluation. At 6 months, we planned to interview up to 30 participants and 20 helpers and at 12 months up to 20 participants. Participants were purposively sampled to include variation in level of app/website use, age and gender. Semi-structured interview guides were used to explore participant insights related to acceptability of the outcome measures, acceptability and usability of the app and website, impact of the intervention on behaviour, support received from helpers and barriers to use [19]. Helper interviews focussed on intervention acceptability, the guidance provided for being a helper, types of support provided to their friend, challenges and changes in their own health behaviour as a result of being a helper. We also asked study participants about the acceptability of the outcome measures and potential contamination. Interviews were audio recorded and transcribed verbatim.

### Economic evaluation

The following resource use items were collected: (i) intervention costs (fixed, variable and likely future annual estimates), (ii) primary care services provided in the NHS (e.g. GP visits, practice nurse), (iii) secondary care services provided in the NHS (e.g. A&E attendances, hospital stay) and (iv) personal costs (e.g. household income spent on food, drinks and lifestyle activities). Medication use at baseline and follow-up was also recorded. Mean group costs were calculated by attaching the unit costs to frequency of resource use per group participant [38]. Median and ranges for resource use quantities and costs are reported. The feasibility of using the EuroQol EQ5D-3L [24] instrument and the ICECAP-A [25] instrument as a means of capturing any short term effects on health-related quality of life or capability wellbeing was assessed. This involved exploring data completeness and response rates. Participant responses for both instruments were converted to a utility score using the value set elicited from UK general population.

### Main analyses

Baseline characteristics were summarised. Feasibility measures were the primary focus of the analysis. Rates of recruitment, randomisation and retention at 12 months follow-up, were calculated with 95% confidence intervals. Usage data for the app and website was explored, and a range of summary measures presented.

Exploratory outcomes were analysed using linear regression models, with randomised group, the baseline measurement of the outcome, age, gender and BMI as predictor variables. Model residuals were assessed for normality, and where necessary, outcome measures (at follow-up and at baseline) were transformed to improve model fit. All analyses were conducted under intention to treat principles. Complete case analysis was used, unless more than 20% of cases were lost due to missing data, in which case multiple imputation was also performed. These analyses were exploratory and underpowered, so no formal hypothesis testing was performed. *p* values are presented for descriptive purposes, as a guide to the interpretation of the magnitude of reported associations. Effect sizes were calculated by dividing intervention effect estimates by the pooled standard deviation of the change from baseline in the outcome measure, and reported in line with CONSORT guidelines for reporting feasibility and pilot studies [39].

### Health economic analyses

The health economics cost data were analysed as follows: Resource use data were summarised and described using mean values and variation around these estimates. Key fixed and variable costs of developing the intervention were described and summarised. EQ5D-3 L [24] and ICECAP-A [25] outcome data were reported by within-attribute response rates, mean values and associated variance. Within-trial economic analyses were performed using STATA 12.0 (StataCorp, TX, USA).

### Qualitative analyses

Qualitative data were analysed by two researchers who independently coded using thematic analysis [40, 41]. Twenty percent of the interviews were double coded by two researchers who resolved disagreements by discussion. The coding framework was discussed between the researchers and also within the wider study team to finalise the themes and sub-themes. The results of the qualitative analyses were combined with intervention usage data to explore and refine the HelpMeDoIt! programme theory, in order to better understand the mechanisms and key contextual factors to consider when refining the intervention and evaluation design.

## Results

**Fig. 2:**
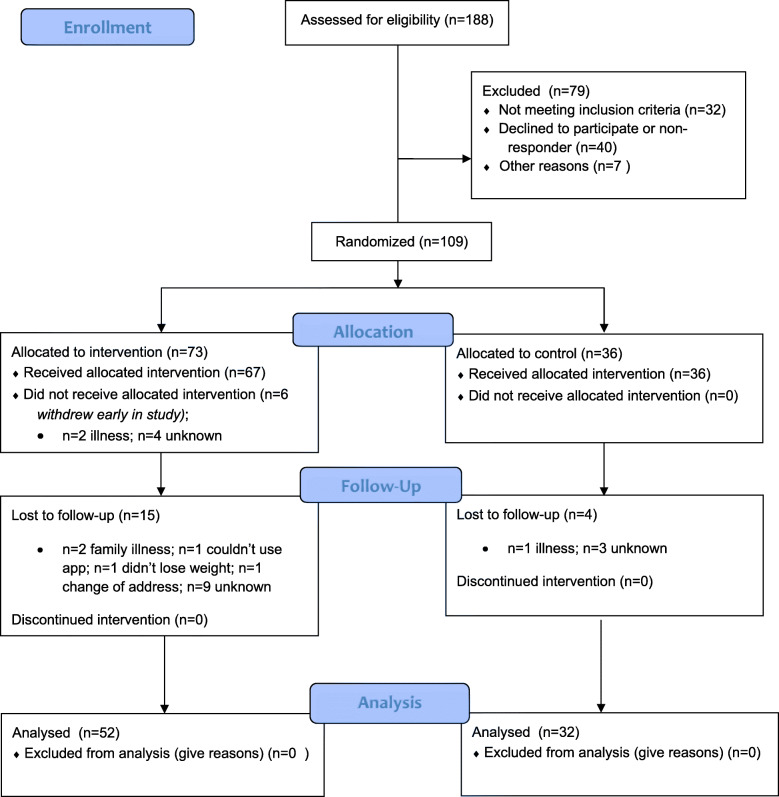
The CONSORT diagram for the HelpMeDoIt! study [15]

109 participants were recruited to the HelpMeDoIt! trial (Fig. 2) and randomised 2:1 to the intervention (*n* = 73) and control group (*n* = 36). Baseline characteristics included 69.7% (*n* = 73) women, mean age of 47 (range 25–68), mean BMI 37.6 kg/m^2^ and 36% were from the highest quintile (most deprived) of socioeconomic deprivation (Table 3) [15]. Key findings are presented below, with additional detailed findings published in our monograph [15].

**Table 3:** Baseline characteristics of randomised participants [15]

		All	Intervention	Control
*N*		109	73	36
Participant age (years)	Mean (SD)	47.3 (10.7)	46.2 (10.6)	49.4 (10.7)
Participant sex	*N* (%) Female	76 (69.7)	49 (67.1)	27 (75.0)
	*N* (%) Male	33 (30.3)	24 (32.9)	9 (25.0)
SIMD quintile^a^	*N* (%) Q1—most deprived	36 (36.4)	25 (37.9)	11 (33.3)
	*N* (%) Q2	21 (21.2)	15 (22.7)	6 (18.2)
	*N* (%) Q3	13 (13.1)	7 (10.6)	6 (18.2)
	*N* (%) Q4	16 (16.2)	10 (15.2)	6 (18.2)
	*N* (%) Q5—least deprived	13 (13.1)	9 (13.6)	4 (12.1)
Marital status^b^	*N* (%) Living with partner	61 (59.2)	41 (60.3)	20 (57.1)
	*N* (%) Single	42 (40.8)	27 (39.7)	15 (42.9)
Ethnicity	*N* (%) White British/Irish	91 (84.3)	57 (79.2)	34 (94.4)
	*N* (%) White Other	6 (5.6)	5 (6.9)	1 (2.8)
	*N* (%) Indian	2 (1.9)	1 (1.4)	1 (2.8)
	*N* (%) Pakistani	2 (1.9)	2 (2.8)	0 (0.0)
	*N* (%) Chinese	1 (0.9)	1 (1.4)	0 (0.0)
	*N* (%) Other	6 (5.6)	6 (8.3)	0 (0.0)
Education^c^	*N* (%) Higher education	64 (61.5)	47 (67.1)	17 (50.0)
	*N* (%) Other	40 (38.5)	23 (32.9)	17 (50.0)
Employment	*N* (%) Employee	86 (78.9)	57 (78.1)	29 (80.6)
	*N* (%) Self-employed	16 (14.7)	12 (16.4)	4 (11.1)
	*N* (%) Not employed	7 (6.4)	4 (5.5)	3 (8.3)
Access to computer at home	*N* (%)	103 (95.4)	68 (94.4)	35 (97.2)
Use internet every day	*N* (%)	105 (97.2)	69 (95.8)	36 (100.0)
Weight (kg)	Mean (SD)	104.6 (20.7)	105.7 (21.4)	102.2 (19.4)
BMI (kg/m^2^)	Mean (SD)	37.6 (5.9)	37.8 (6.0)	37.1 (5.7)
Waist circum. (cm)	Mean (SD)	114.1 (14.7)	113.9 (15.4)	114.6 (13.2)
Hip circum. (cm)	Mean (SD)	124.3 (13.4)	125.0 (14.2)	122.7 (11.7)

^a^SIMD = Scottish Index of Multiple Deprivation

^b^Living with partner = married/civil partnership/cohabiting; Single = single/widowed/divorced

^c^Higher education = higher degree/first degree/certificate/diploma; Other = A or AS/O levels/other

### Progression criteria

The pre-specified progression criteria (Table 1) were achieved [15]. It was feasible to recruit and retain participants in the trial (progression criteria 1–3). Our target sample size was 120 participants. Our recruitment was hampered by a 2-month delay in gaining governance approvals. We recruited 109 participants over a 6-month period (91% of our target). In order to keep within the study timeline we had to stop recruitment at 6 months. This was just short of our target which would likely have been achieved if part of our recruitment had avoided the summer holiday months. The slightly smaller sample size was not an issue in terms of the study aims, in order to assess the acceptability and feasibility of the intervention and evaluation methods the sample size we recruited was sufficient. At 12 months we achieved a follow-up rate of 77.1% (84 of 109 participants). Follow-up rates were different between intervention and control groups (71% and 89%, respectively). We developed an intervention that was feasible to deliver and acceptable to helpers and participants (see the ‘Process evaluation findings’ section data) (progression criteria 4). Two thirds of intervention participants (including those who withdrew from the study) visited the app twice or more, and 52% visited three times or more (progression criteria 5). See the ‘Process evaluation findings’ for further usage data. Data collection methods were feasible to use except for the 24-h multiple pass recall dietary measure, which was poorly completed at baseline and therefore not used at follow-up, and issues with obtaining valid accelerometry data (progression criteria 7). Barriers and challenges to implementation have been planned for and are surmountable (progression criteria 6). This is discussed further below.

### Process evaluation findings

A summary of the key findings from the process evaluation is given below with further details published elsewhere [15]. Interviews were conducted with 35 individuals (22 participants and 9 helpers at 6 months and 4 participants at 12 months). Most study participants interviewed were positive and engaged with the HelpMeDoIt! intervention. All study participants used a helper to support them with weight loss. Many of these helpers were nominated through the app; however, for some participants, this was done outside of the app. Social support was a key element, with helpers providing emotional, informational and instrumental support to participants. The emotional support and encouragement from helpers were seen as key. Many participants set goals via the app (mean 13.1 range 0.143) for healthy eating, physical activity and other behaviours. Participants reported monitoring their progress toward goals and also using other apps for self-monitoring. Sixty-one percent of the 954 goals created by participants were completed. Participants who were most successful at losing weight had varied and good social support and were positive about the goal setting and self-monitoring.

Helpers described how they enjoyed supporting their friend with their weight loss goals. They reported receiving mutual support with their own lifestyle goals and that they were more motivated to eat well and be active. Few helpers used the app, they reported technical difficulties, lacking confidence with smartphones, or preferring to support their friend outside of the app, e.g. face-to-face interactions. They believed their support contributed to their friend’s motivation to make healthy changes.

Interview findings helped inform refinement of the programme theory and logic model. Motivation was identified as a key mediator influencing behaviour and encouragement from the helpers was important to boost motivation. Participants reported positive lifestyle changes in both their helpers and their broader social network. Contextual factors like mood or significant life changes were reported as influencing participants’ engagement with the intervention. Factors highlighted for consideration in future work included difficulty asking friends/relatives for support, lack of available support, social and group norms related to food and exercise and personal barriers to lifestyle, such as motivation.

Despite a 3-month testing phase, there were initial technical issues with the app. The majority of dissatisfaction and barriers to use were related to these issues. The app underwent a ‘rebuild’ which resolved the software problems. Participants who used the app most frequently, once the technical issues were resolved, provided the most positive feedback via both qualitative and quantitative measures. Of the 54 (74%) participants who downloaded the app, 48 (89%) used it twice or more. In total, 45 helpers were invited, ranging from 1 to 8 helpers per participant. Of the 45 invited helpers, 25 (56%) accepted the invitation and downloaded the app. Most helpers did not engage with the app on a frequent basis. However, interview data indicated that helpers were sometimes unclear how to use the app to help their family or friend, with many providing support through face-to-face interactions instead. While participants and helpers did not access the website regularly, it is likely that one or two visits would be enough to get the information needed. The app also delivered the key information from the website via push notifications and daily messages.

Although not part of the process evaluation, we asked participants about the trial methods. They were positive about the evaluation methods, such as the data collection measures and retention strategies and there was no evidence of contamination in the data.

### Exploratory outcomes

The feasibility trial was not powered to detect statistically significant changes, but to explore the feasibility and sensitivity of measures for use in a definitive trial. Three outcomes were assessed: BMI, physical activity and diet. BMI was successfully measured in 98% of the sample (82% objectively and 16% via self-report) and diet (DINE questionnaire) in 96% (81 of 84). Physical activity data were successfully collected via the self-report 7-day physical activity recall from 96% of participants. However, objective accelerometry was only available from 46% of participants. The secondary outcomes were feasible and acceptable to use.

Objective physical activity data showed moderate to large effect size estimates across several measures, particularly the daily step count and sedentary time (Table 4). There was no evidence to suggest that self-report physical activity was different between those who did and did not provide valid accelerometry data, thereby increasing confidence in these results. However, these outcomes were poorly completed with only 24 in the intervention arm and 15 in the control providing valid data. Both methods suggested a decrease in physical activity from baseline, with accelerometry showing a decrease in both groups for MVPA, and self-report showing a decrease in both groups for energy expenditure. With regard to the diet scores, there was low power and no consistent patterns in terms of between-group differences (Table 4).

**Table 4:** Measures of BMI, physical activity and dietary outcome (DINE) at baseline and at 12-month follow-up for the subset of participants providing data at both time points [15]

	*N*	Baseline	12 months	Change	Between-group difference (Int^*n*^ − Control)		
						Estimate (95% CI)	ES (95% CI)
BMI (kg/m2)							
Con	32	36.9 (5.7)	36.0 (6.3)	− 0.9 (3.3)	Unadj.	− 0.3 (− 1.5 to 0.9)	− 0.11 (− 0.56 to 0.33)
Int	50	36.9 (5.3)	35.7 (5.4)	− 1.2 (2.4)	Adj.	− 0.2 (− 1.4 to 1.0)	− 0.08 (− 0.52 to 0.37)
MVPA time as % of wear time (from activity monitor)*							
Con	15	4.1 (2.3)	3.5 (2.3)	− 0.6 (1.0)	Unadj.	− 0.3 (− 2.2, 1.7)	− 0.08 (− 0.73, 0.56)
Int	24	6.5 (4.0)	5.6 (2.5)	− 0.9 (3.8)	Adj.	1.3 (− 0.1, 2.7)	0.44 (− 0.02, 0.90)
Average daily MVPA time (min, from activity monitor)							
Con	15	35.5 (19.4)	31.3 (20.3)	− 4.3 (10.5)	Unadj.	− 4.2 (− 21.1, 12.7)	− 0.16 (− 0.81, 0.48)
Int	24	54.8 (34.3)	46.3 (20.2)	− 8.5 (32.2)	Adj.	9.3 (− 2.3, 20.9)	0.35 (− 0.09, 0.80)
Average daily sedentary time (min, from activity monitor)							
Con	15	661.7 (138.2)	703.4 (166.6)	41.7 (83.5)	Unadj.	− 52.9 (− 104.4, − 1.4)	− 0.66 (− 1.31, − 0.02)
Int	24	642.7 (94.0)	631.5 (82.8)	− 11.2 (77.6)	Adj.	− 60.8 (− 110.5, − 11.0)	− 0.76 (− 1.38, − 0.14)
Average daily step count (from activity monitor)							
Con	15	5650 (1526)	5335 (1844)	− 315 (1130)	Unadj.	43 (− 1876, 1963)	0.01 (− 0.63, 0.66)
Int	24	7232 (3712)	6960 (2568)	− 272 (3669)	Adj.	1187 (− 180, 2555)	0.40 (− 0.06, 0.86)
Average daily energy expenditure (kcal/day, self-report)							
Con	32	3879 (1121)	3606 (750)	− 273 (828)	Unadj.	113 (− 179, 404)	0.17 (− 0.27, 0.62)
Int	49	3717 (715)	3557 (779)	− 160 (502)	Adj.	62 (− 180, 304)	0.09 (− 0.28, 0.47)
Average daily energy expenditure per kg body weight (kcal/kg/day, self-report)							
Con	32	38.2 (7.1)	36.7 (4.1)	− 1.6 (6.5)	Unadj.	1.0 (− 1.6, 3.6)	0.17 (− 0.27, 0.62)
Int	49	37.2 (4.9)	36.7 (3.7)	− 0.6 (5.3)	Adj.	0.0 (− 1.7, 1.6)	0.00 (− 0.29, 0.28)
Fibre score (score < 30 = low fibre)							
Con	32	18.6 (10.6)	19.2 (11.6)	0.6 (12.6)	Unadj.	− 4.1 (− 9.2, 0.9)	− 0.36 (− 0.81, 0.08)
Int	49	19.8 (9.7)	16.3 (11.8)	− 3.6 (10.6)	Adj.	− 3.3 (− 8.1, 1.5)	− 0.29 (− 0.71, 0.13)
Fat score (score < 30 = low fat)							
Con	32	27.1 (12.6)	22.9 (9.6)	− 4.1 (10.8)	Unadj.	− 1.4 (− 6.4, 3.6)	− 0.13 (− 0.57, 0.32)
Int	49	28.4 (11.6)	22.9 (12.7)	− 5.5 (11.4)	Adj.	− 0.4 (− 4.8, 4.0)	− 0.04 (− 0.43, 0.36)
Healthy eating score (score = fibre − fat; negative score indicates unhealthy diet)							
Con	32	− 8.5 (16.1)	− 3.8 (13.2)	4.7 (16.6)	Unadj.	− 2.7 (− 8.9, 3.4)	− 0.20 (− 0.64, 0.25)
Int	49	− 8.6 (13.3)	− 6.6 (12.9)	2.0 (11.7)	Adj.	− 2.9 (− 8.0, 2.2)	− 0.21 (− 0.58, 0.16)
Unsaturated fat score (score 6–9 = moderate unsaturated fat intake)							
Con	32	9.3 (1.8)	8.2 (3.7)	− 1.1 (3.5)	Unadj.	− 2.3 (− 4.3, − 0.3)	− 0.45 (− 0.85, − 0.06)
Int	49	9.2 (2.2)	5.8 (5.0)	− 3.3 (5.6)	Adj.	− 2.2 (− 4.0, − 0.4)	− 0.44 (− 0.80, − 0.07)
Fruit and vegetable score (score = portions per day)							
Con	32	5.0 (3.4)	4.9 (2.9)	− 0.1 (3.4)	Unadj.	0.7 (− 0.6, 1.9)	0.24 (− 0.21, 0.69)
Int	49	4.7 (2.6)	5.2 (2.5)	0.5 (2.2)	Adj.	0.4 (− 0.6, 1.5)	0.16 (− 0.23, 0.55)
Fizzy juice score (score = cans of juice per day)							
Con	32	0.2 (0.6)	0.3 (0.9)	0.1 (0.4)	Non-parametric test^a^		
Int	49	0.0 (0.0)	0.2 (0.6)	0.2 (0.6)			
Sugar score (score = tsps of sugar per day)							
Con	32	0.9 (5.3)	0.8 (3.5)	− 0.2 (1.9)	Non-parametric test^a^		
Int	49	0.5 (1.3)	0.3 (0.8)	− 0.2 (0.9)			

Models adjusted for baseline value, age, gender and high BMI (> 40)

*MVPA, moderate or vigorous physical activity

^a^Not suitable for linear regression modelling. Change from baseline compared between groups using Wilcoxon-Mann-Whitney test. Median difference with 95% CI reported

Both groups showed similar reductions in BMI during the study of − 1.2 kg/m^2^ (SD 2.4) in the intervention group, and − 0.9 kg/m^2^ (SD 3.3) in the control group. Mean kg weight loss for intervention participants was − 3.3 kg (SD 6.5) compared with − 2.5 kg (SD 9.3) for control participants. Mean % weight loss for intervention participants was − 3.2% (SD 6.2%), and − 2.3% (SD 8.7%) for control participants (Table 4).

Sixty-six percent of 50 intervention group participants for whom weight was measured at both baseline and follow-up had lost weight, compared to 53% of 32 participants in the control group (*p* = 0.26). These analyses were exploratory and underpowered to detect effects. However, for the key weight-related outcomes of interest, the confidence intervals were generally wide and therefore consistent with clinically relevant benefits.

### Economic evaluation

Total study intervention costs (fixed and variable) included app development costs of £60,000 and incentive/retention payments of £4360 (vouchers, newsletters). Depending upon throughout and lifespan, ‘per participant’ cost will vary by study. The resource use patterns were similar across groups; the main items of resource use were GP, practice nurse, physiotherapist, A&E and hospitalisations. The cost of lifestyle activities in the 3 months prior to follow-up was in the range £50–55. Mean weekly cost of food and drink at follow-up was in the range of £75–100 across both groups with most food expenditure on groceries followed by meals out, take away and alcohol spend. This pattern was the same across both arms, at baseline and follow-up. Both QOL and capability wellbeing questionnaires were completed by participants at baseline and 12 months follow-up. At 12 months, 78 participants completed the EQ5D-3L [24], ICECAP-A [25] and expenditure on food purchases. Only 69 provided data on health, social and personal resource use. This was because a few participants at risk of dropping out of the study were offered a ‘minimum data set’ that omitted the additional questions linked to resource use. The EQ5D and associated visual analogue scale results revealed the typical ‘healthy population’ values of around 0.7–0.8 and revealed expected variation in values between the measures. There were no implausible data for the EQ5D_3L. Full economic findings, including medication use and mean group costs, can be found in our published monograph [15].

Overall, the results of the economic evaluation feasibility study showed that the questionnaires designed for measuring resource use, lifestyle, grocery and alcohol spend, health-related quality of life and capability would be suitable for inclusion in a full study with some minor re-design of the resource use questions.

There were no serious adverse events reported during the study.

## Discussion

Family and friends are likely to be a key influence on weight-related health behaviours [4, 5], since these are the people whom we are closest to, spend most time with and with whom we are likely to eat and exercise with. For these reasons, they are likely important not only for initiation of behaviour change, but also for longer term maintenance. Although other apps/websites develop user communities that offer social support, they are unlikely to have such a large and sustained influence as family and friends. This study assessed the feasibility and acceptability and impact of a novel, theory-informed weight loss intervention that combined evidence-based behaviour change techniques with mobilising social support from a participant’s close social network.

Overall, the study findings were positive indicating the intervention and trial methods were feasible and acceptable and both the qualitative and quantitative results indicated the intervention had potential. Most effect size estimates had confidence intervals that included 0.5 in favour of the intervention, which would generally be considered a moderate effect size for a low-cost intervention of this nature. Although MVPA and energy expenditure decreased in both groups, participants were relatively active at baseline, with the intervention arm achieving an average of 50 min and controls an average of 36 min of moderate to vigorous physical activity per day. It should also be noted that studies have found that people with obesity are likely to over-estimate their physical activity using self-report measures [42]. In addition, all key progression criteria were achieved, recruitment rates were adequate (91% of target), retention was good (77.1% at 12-months) and engagement with the intervention was acceptable.

Qualitative data supported the programme theory of the intervention (Fig. 1) [15], since the app facilitated engaging support from people that participants already knew to help their weight loss attempts. The findings supported the key role of social support from existing social networks. Motivation, goal setting and self-monitoring were supported as core elements of the programme theory. The app was a catalyst to engaging social support either via the app or outside of the app. It is likely that a number of participants in the study who enrolled or downloaded the app did not engage or benefit from the intervention. However, this does not undermine the potential reach and cost effectiveness, as there is some evidence that the app may be useful for some individuals and this is a low-cost way to facilitate social support from close social networks, which is known to be effective in assisting and maintaining behaviour change.

The low engagement with the website by both participants and helpers suggests a need for better signposting, linking to website information within the app, or perhaps that the website is not needed. There was also low engagement of helpers via the app. Technical issues with the app in the early stages of the trial may have led to participants disengaging with the app and the trial. Adherence to the intervention was superior to that seen in general app usage [43] and similar to that seen in other studies testing behaviour change apps [44, 45]. An expert international workshop concluded that engagement with apps is complex and is more than how often a person simply uses an app [46]. Continued engagement with a digital intervention (app/website) is not always needed for behaviour change, as initial exposure to a digital intervention could be enough to kick start a process of establishing new skills and habits [46]. This is particularly relevant to HelpMeDoIt!, where a brief interaction with the website or app could lead to the engagement of significant and sustained social support from existing, stable and durable social resources. The effects of this support on resultant behaviour are not dependent on further app use.

The study had a number of key strengths. The intervention was developed with substantial input from potential users and was developed using recommended frameworks for developing complex interventions and digital interventions. The intervention was theory-based and informed by current evidence regarding successful behaviour change techniques for weight loss. We used a multiple methods approach and were able to triangulate these data to strengthen the internal and external validity of the findings.

The feasibility trial used rigorous methods for data collection and analyses. Objective measures were used to assess weight, BMI and physical activity, and self-report measures were chosen based on previous evidence of validity and reliability. The study also included measures of quality of life and capability wellbeing and collected cost data to inform a future cost-effectiveness analysis. We recruited a clinically important sample with a mean BMI of 37.6 kg/m^2^ (SD 5.9). We were particularly successful in recruiting participants from lower socioeconomic groups with over a third from the highest quintile of deprivation in Scotland and a further 21% from the next most deprived quintile. This is important as research has shown that it is often difficult to recruit and engage participants from lower socioeconomic groups in research [47] and many current interventions widen inequalities [48]. Finally, the qualitative methods were robust, and the large number of interviews gave extensive, in-depth accounts of the experiences of both the study participants and their helpers. The findings from the process evaluation informed refinements of the programme theory, logic model and trial methods in anticipation of a future effectiveness trial.

Limitations of the study included ethical constraints during the stage 2 follow-up, which meant that we were unable to invite helpers directly to be interviewed. Because of this, the number of helper interviews was smaller than anticipated. Only about half the participants had complete accelerometry data. A wrist worn accelerometer may have had better compliance. In other similar studies, strategies like vouchers for return of the accelerometers have led to improved adherence and return rates, so vouchers combined with other methods could significantly improve return rates in a future trial [49, 50].

While the aim of this research was to develop an intervention that maximised effect size and participation, an intervention like this could have high reach and therefore tolerate small effects and low usage overall while remaining cost-effective. For some people, meaningful engagement will be catalysed by the app for whom the effects should be worthwhile. It will also be important to consider the contribution of such mHealth approaches within the wider ecological public health context for weight management and sustained weight reduction. If the intervention was found to be effective in a larger trial, then HelpMeDoIt! may have the potential to deliver a low-cost, high-reach intervention for adults with obesity, including those in socioeconomically disadvantaged groups. It could be used as a complementary intervention used alongside other health care or lifestyle services. HelpMeDoIt! may have potential to positively influence the lifestyle of individuals in a participants’ broader social network. This approach to mobilising social support for health behaviour change could be used in other lifestyle behaviours or used as part of other app-based interventions. Key areas of future work include further exploration of the key mechanisms of change as well as the motivation and engagement of helpers in relation to providing social support to participants and then after some refinement, assessment of the effectiveness and cost-effectiveness of the HelpMeDoIt! intervention.

## Data Availability

The datasets used and/or analysed during the current study are available from the corresponding author on reasonable request.
